# AI-supported case-based learning in medical education: a comprehensive scoping review

**DOI:** 10.3389/fmed.2026.1798097

**Published:** 2026-03-25

**Authors:** Syed Hani Abidi, Joseph Almazan, Olaoluwa Fabiyi, Fatin Zehra, Muhammad Tariq

**Affiliations:** 1 Department of Biomedical Science, School of Medicine, Nazarbayev University, Astana, Kazakhstan; 2 Department of Medicine, School of Medicine, Nazarbayev University, Astana, Kazakhstan; 3 College of Education and Human Development, Louisiana State University Shreveport, Shreveport, LA, United States; 4 Department of Medicine, Aga Khan University, Karachi, Pakistan

**Keywords:** case-based learning, generative AI, learning, teaching, assessment

## Abstract

**Introduction:**

Recent literature indicates that generative artificial intelligence (GenAI) is also being integrated into case-based learning (CBL) through activities such as clinical case generation, clinical reasoning support, and structured feedback. However, the evidence about GenAI’s role in CBL remains fragmented. Given the diverse nature of available GenAI-CBL studies, we conducted a comprehensive scoping review to map and synthesize the evidence in this area, highlighting key themes, outcomes, challenges, and limitations to inform future research, curriculum development, and policies.

**Method:**

A comprehensive search was performed across multidisciplinary databases, including PubMed, ERIC, Scopus, Web of Science, and Google Scholar, covering publications from 2019 to 2025. Title and abstract screening, followed by full-text review and data extraction, were conducted independently by two reviewers using predefined eligibility criteria. The data synthesis involved thematic analysis to create an evidence map of GenAI-supported case-based and case-anchored learning in medical education.

**Results:**

The findings were organized into six key themes that showcase the role of GenAI in enhancing case-based learning, covering areas such as clinical reasoning and contextual thinking; efficient and scalable case creation; learner engagement, motivation, and perceived usefulness; accuracy, reliability, and ethical issues; faculty adaptation and pedagogical integration; and hybrid and reflective learning methods.

**Conclusion:**

Overall, the evidence indicates that GenAI can effectively support CBL in medical education, especially during early and intermediate stages. It also highlights the ongoing importance of faculty oversight and the need for further research to address advanced clinical judgment and ethical reasoning.

**Systematic review registration:**

https://doi.org/10.17605/OSF.IO/28E3G, identifier (28E3G).

## Introduction

Medical education is a structured process that prepares physicians to deliver high-quality, patient-centered, and socially accountable care (1). This also includes continued professional development to maintain and enhance competence, integrate new evidence and technologies into practice, and ensure their care remains aligned with evolving standards and patient needs (2). Medical education primarily emphasizes outcomes that evaluate students’ specific behaviors, knowledge, and skills, rather than merely providing instruction and training. These outcomes encompass patient-centered care, professional ethics, clinical competence, and effective teamwork, all aligned with the principles of the Competency-Based Medical Education (CBME) framework (3).

Since CBME focuses on demonstrating professional behavior, competence, and knowledge, the teaching/learning strategies should be more learner-centered and interactive rather than relying on passive lectures. Instructional strategies such as problem-based, team-based, and case-based learning effectively support competence-based outcomes (4). Among these, case-based learning (CBL) provides a flexible, structured teaching approach that embodies the principles of CBME by situating learning in real-world contexts that require practical skills and decision-making (5). CBL is a guided inquiry approach that utilizes structured case-based scenarios to organize and implement knowledge and training. It is grounded in established learning science principles, including retrieval practice, contextualized application of knowledge, and transfer across variable scenarios, which provide a strong theoretical foundation for its use (6). Evidence suggests that CBL facilitates the translation of clinical theories and conceptual frameworks into practical decision-making, thereby supporting patient care and safety (7). For example, McLean (8) demonstrated that CBL enhances learners’ ability to apply theoretical knowledge in clinical practice. Similarly, Ali et al. (9) reported that the implementation of CBL was associated with improved independent learning and increased student engagement, with reported gains exceeding 70%. Despite these demonstrated benefits, the widespread and sustained use of CBL is constrained by the practical challenge of developing and maintaining a continuous supply of diverse, complex, and clinically authentic cases, a process that is time-intensive and often dependent on substantial faculty effort and interdisciplinary collaboration (6).

Generative artificial intelligence (GenAI) offers a scalable method to produce diverse, realistic, and clinically relevant scenarios on demand, combining the scientific benefits of CBL with curriculum requirements (10). Additionally, integrating GenAI with CBL enables instructors to provide personalized tutoring and develop more effective assessments tailored to learners’ individual needs (11). The GenAI-CBL merger is important but underexplored, with few studies examining its use in generating clinical cases or exploring healthcare professionalism through simulation scenarios (11, 12). The GenAI–CBL merger also aligns with cognitive load theory, which holds that learning is most effective when the cognitive load on a student’s working memory is balanced (13). Since case-based learning can be inherently demanding and impose a high cognitive load, GenAI can be effectively employed to manage and reduce this load. For instance, GenAI can clarify technical terms, break down complex cases into more manageable parts, and help learners understand the connections among different case components, freeing up mental resources for in-depth analysis, clinical reasoning, and knowledge absorption (14).

Despite these benefits, evidence supporting the efficacy of GenAI in teaching, learning, assessment, and, especially, skills acquisition remains limited (15). Specifically, the literature on GenAI-supported CBL is heterogeneous, characterized by inconsistent terminology, limited methodological comparability, a lack of scalable outcome measures, and a predominance of descriptive reports. Consequently, a distinct, theory-informed synthesis that elucidates how GenAI aligns with learning principles and whether it substantively improves CBL outcomes is presently lacking. To address this conceptual and methodological gap, we conducted a scoping review rather than a systematic review to map existing evidence and characterize the current state of research on GenAI in CBL (16).

This comprehensive scoping review maps and synthesizes the evidence in this area, highlighting key themes, outcomes, challenges, and limitations to inform future research, curriculum development, and policy.

## Objectives

1. To map the current landscape of how generative AI tools are used to support case-based learning in undergraduate and postgraduate medical education.
2. To identify and categorize the instructional designs, learning environments, and modalities in which generative AI is integrated into case-based learning.
3. To analyze reported educational outcomes, learner engagement, and skill development resulting from AI-supported case-based learning interventions.
4. To explore perceptions, acceptability, and ethical issues of using generative AI among medical students and educators in case-based learning settings.
5. To identify research gaps and methodological limitations in the current literature to inform future empirical studies and the development of educational interventions.

## Methods

This scoping review was conducted between August to December 2025, following the Joanna Briggs Institute (JBI) framework and the Preferred Reporting Items for Systematic Review and Meta-Analysis extension for Scoping Reviews (PRISMA-ScR) guidelines (17). The PCC framework was used, with the *Population* defined as medical students and educators, the *Concept* as the use of generative artificial intelligence in CBL and closely related learning activities grounded in clinical scenarios, and the *Context* as medical education settings.

In accordance with open science practices, the study protocol was registered on Open Science Framework (OSF) for transparency (DOI: https://doi.org/10.17605/OSF.IO/28E3G) and released as a peer-reviewed publication prior to data analysis (18).

### Search strategy

A systematic search was conducted in PubMed, ERIC, Scopus, Web of Science, and Google Scholar (grey literature) using the search string: (“ChatGPT” OR “generative AI” OR “large language model*” OR “Claude” OR “Gemini”) AND (“case-based learning” OR “CBL”) AND (“medical education [MeSH]” OR “medical student*” OR “medical teach*”). Filters were applied for English-language publications from 2019 to 2025. For Google Scholar, 15 pages (*n* = 150) were screened.

### Eligibility criteria

Studies were included if they: (1) involved medical learners or educators; (2) examined the use of generative AI in CBL within medical education; (3) were published in English between 2019 and 2025 (reflecting the period of rapid expansion and educational adoption of large language models and conversational generative AI systems) and (4) had full-text access, defined as the availability of the complete published article online through either open-access sources or authorized platforms such as publisher websites and institutional library subscriptions.

Eligible study designs included qualitative, quantitative, mixed methods, pilot, conceptual framework, and commentary articles. Exclusion criteria were studies focused solely on non-medical health professions (unless explicitly interprofessional), AI applications that did not involve generative models, and educational uses without clear case, problem, or assessment-anchored learning components.

### Study selection

Search results were exported to Zotero (19), and duplicates were removed manually. Two independent reviewers screened titles and abstracts, followed by full-text assessment of potentially eligible articles. Disagreements were resolved through discussion, with a third reviewer consulted to reach a consensus. The third reviewer also supervised the screening and final study selection to ensure methodological rigor. Given the broad and rapidly expanding use of generative AI across multiple disciplines, the initial search strategy intentionally prioritized sensitivity over specificity, resulting in a high number of retrieved records. To minimize the risk of missing relevant studies, screening was conducted in multiple stages using predefined inclusion and exclusion criteria. A large proportion of records were excluded at the title and abstract stage because they focused on non-medical disciplines, non-educational applications of generative AI, or GenAI uses unrelated to case-based learning. Full-text screening was applied conservatively, with inclusion decisions requiring clear alignment with both GenAI use and a case-based learning framework. Independent dual screening and third-reviewer oversight were used to reduce the likelihood of erroneous exclusion.

For the purposes of this review, CBL was operationally defined broadly to include structured case-based, case-anchored, and case-driven educational activities in which learners engaged with clinical scenarios to support reasoning, decision-making, or reflective learning. This included studies where cases were used for instructional, formative, or assessment purposes, provided that learners actively interacted with the case content and received explanatory feedback or reasoning support. Topic-focused case applications and assessment-oriented case vignettes were therefore included when they functioned as learning tools rather than isolated knowledge tests.

### Data extraction and evidence synthesis

Data extraction was conducted using a standardized extraction form that documented study details, participants’ characteristics, the generative AI tool, CBL features, and reported outcomes. A second reviewer cross-checked the extracted data for accuracy. Findings were summarized descriptively to outline the existing evidence on GenAI-supported CBL in medical education. An inductive thematic analysis was performed, involving familiarization with the data, initial open coding, and repeated grouping of codes into broader themes. Coding and theme development were performed independently by two reviewers, and discrepancies were resolved through discussion to reach consensus. Additionally, the Dreyfus model of skill acquisition (20, 21) was used as an interpretive lens to contextualize the themes identified in the scoping review. Here, we examined how each theme (generated in this scoping review) aligned with typical learner behaviors across the five Dreyfus stages (*Novice, Advanced Beginners, Competent, Proficient*, and *Expert*). This alignment was based on conceptual considerations rather than detailed analysis.

## Results

**Figure 1:**
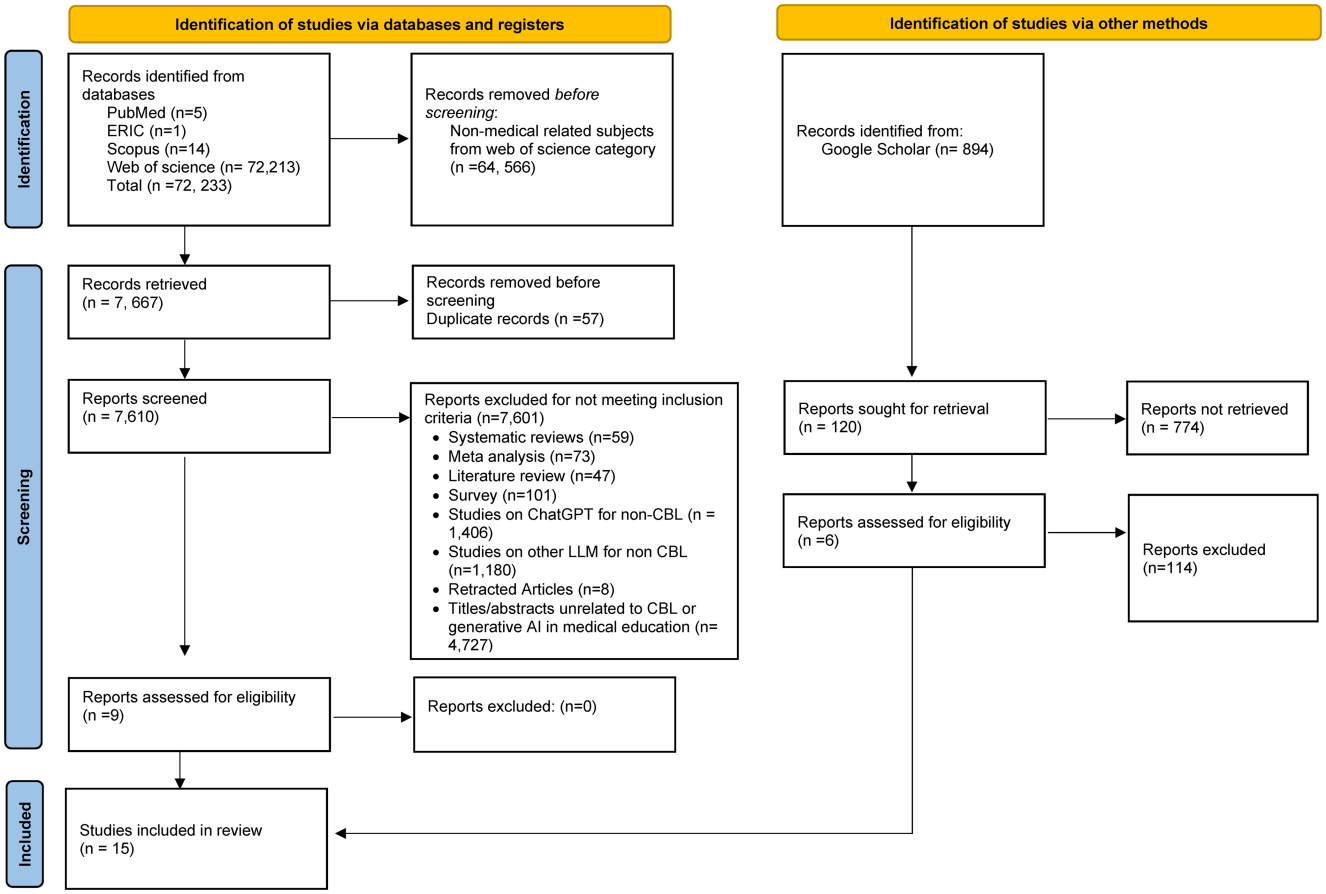
PRISMA flow diagram of study selection.

The screening and selection process, as well as the inclusion and exclusion criteria for studies, is detailed in the PRISMA flowchart (Figure 1). The initial search across PubMed, ERIC, Scopus, and Web of Science yielded 72,233 records. An additional 894 records were found via Google Scholar. After screening titles and abstracts, 64,566 non-medical records were excluded based on Web of Science subject categories, and 57 duplicates were removed. This process left 7,610 records for further evaluation. Studies not fitting the inclusion criteria, such as systematic reviews, meta-analyses, narrative reviews outside the scope of generative AI-supported case-based learning in medical education, as well as surveys, studies on large language models for non-CBL purposes, retracted articles, and titles/abstracts not related to CBL or generative AI, were excluded. In total, 7,601 records were excluded at this stage, and nine studies were retrieved from databases and six from Google Scholar for full-text screening. Finally, 15 articles published between 2023 and 2025 were included in this study. Some included studies employed topic-specific or assessment-linked case formats; however, these were retained when the case functioned as a learning scaffold that involved reasoning, explanation, or reflective engagement rather than as standalone testing.

The characteristics of shortlisted studies are detailed in Table 1. Across the 15 studies included, participant characteristics varied by study design and educational context, including differences in participant type, sample size, and geographical distribution. Overall, the studies involved predominantly undergraduate medical students (*n* = 446). Smaller cohorts included anesthetic trainees (*n* = 4), hematology residents (*n* = 60), and educators and practicing medical professionals (*n* = 52). Sample sizes varied substantially across studies, ranging from small pilot and feasibility cohorts to larger quasi-experimental and classroom-based comparative studies (Table 1). This variation in participant type and sample size highlights the breadth of interest in applying generative AI across multiple stages of medical education and professional training.

**Table 1:** Characteristics of studies included in the scoping review.

References/country	Aims	Study design	Generative AI used	Findings
Li et al. (22)/China	To explore the impact and limitations of the generative AI (GenAI) tool, specifically Kimi Chat 2.0, in clinical biochemical case-based learning (CBL).	Comparative experimental study	Kimi chat 2.0	The experimental (GenAI) group outperformed the control group in case completion time (2.6 h vs. 5.5 h) and exam score (77.3 ± 4.3 vs. 66.5 ± 5.4, *p* < 0.05). GenAI-based grading demonstrated high reliability and objectivity, aligning with teachers’ evaluation (*p* > 0.05). Students rated GenAI highly for basic knowledge acquisition (9.18/10) and noted limitations in complex clinical reasoning (4.20/10), and innovative thinking (3.90/10).
Hou et al. (23)/China	To evaluate the efficacy of integrating the open-source large language model (LLM) into the problem-based learning (PBL) curriculum for hematology residency training	Quasi-experimental, non-randomized control trial.	DeepSeek	DeepSeek users scored higher on case analysis (4.40 vs. 3.97), understanding of medical concepts (4.33 vs. 3.87), course structure (4.47 vs. 3.93), and clinical reasoning support (4.17 vs. 3.60). Users reported better alignment with learning needs (4.30 vs. 3.83, *p* = 0.007), higher confidence in content accuracy (4.23 vs. 3.50), more trusted feedback (4.47 vs. 4.03), and clearer explanations (4.53 vs. 3.73). Users rated engagement (4.03 vs. 3.53) and motivation for future use (4.47 vs. 3.63) higher than the comparison group.
Zeng et al. (24)/China	To explore the effectiveness of combining traditional teaching methods with ChatGPT-based learning in teaching rare diseases like retinitis pigmentosa (RP).	Quasi-experimental study	ChatGPT	Students using ChatGPT showed higher post-test scores on retinitis pigmentosa content (115.42 ± 22.70 vs. 110.93 ± 29.01). ChatGPT group required much less review time (24.29 ± 12.62 vs. 42.54 ± 20.43 min). ChatGPT users performed better on complex clinical reasoning.
Qian et al. (25)/United States	To examine the delivery and scoring of case-based learning by a GPT-4o, and to compare it is scoring of medical students’ interactions with cases against a panel of faculty expert scorers	Pilot comparative study	ChatGPT 4o	Expert scoring averaged 70.3% (SD = 21.2%, median = 75%), while LLM was higher at 82.2% (SD = 16.1%, median = 82.8%). LLM scores were significantly higher (*p* < 0.01), about 12% above expert ratings (Bland–Altman analysis). After a 10-point calibration, LLM and expert scores no longer differed significantly, indicating closer alignment between the two models.
Lundgren et al. (26)/United States	To translate learnings from previous research into viable means to ensure practitioners’ uptake of information for dealing with uncertainty through case-based learning activities in preparation for the third-year clerkship	Action research	Claude.ai and ChatGPT	Iterative refinement of AI-generated cases improved student engagement and strengthened critical thinking about uncertainty.
Trewren et al. (27)/Australia	To assess whether the LLM could support case-based learning in perioperative medicine with a high degree of accuracy	Single-group feasibility study	ChatGPT 4o	LLM responses showed very high accuracy (99.3%; 543/547) with no hallucinations observed. The LLM added medically correct details, verified by experts, beyond the script in 79 cases (14.4%) and appropriately declined when information was unavailable.
Stretton et al. (28)/Australia	To explore the potential applications, benefits, and limitations of LLM in the development and facilitation of case-based and problem-based learning (CBL/PBL).	Commentary	ChatGPT	GenAI can create realistic, customizable clinical cases that align with learning objectives and enhance student engagement.
Hassoulas et al. (29)/United Kingdom	To evaluate whether integrating emerging technologies into case-based learning for second-year medical students would enhance learning experience, performance, and confidence in clinical and communication skills.	Randomized matched control pilot study	ChatGPT-based system created by SimPat for virtual patient simulation	The TEL-CBL group scored higher on quizzes than the control group (57.96 ± 9.58 vs. 49.75 ± 11.48), with the gap increasing over time, and higher TEL use correlated with better performance (*X*^2^ = 9.89, *p* < 0.05). About 70% of TEL-CBL students felt better prepared for exams compared with peers in the traditional CBL group.
Berbenyuk et al. (30)/United Arab Emirates	To explore the feasibility and educational value of using AI-generated clinical cases in continuing medical education (CME)	Feasibility study	ChatGPT 4o	AI-generated cases were rated as realistic, reflective, and sufficiently complex for healthcare learners, with strong scores across seven domains. Higher ratings observed for evidence-based content (4.03 vs. 3.75, *p* = 0.018), complexity (4.17 vs. 3.75, *p* = 0.004), and coherence (4.17 vs. 3.81, *p* = 0.009).
Oncu et al. (31)/Turkey	To evaluate the impact of ChatGPT-4o on the clinical case management competencies, specifically problem-solving, clinical reasoning, and crisis management of sixth-year medical students.	Mixed method design	ChatGPT 4o	There was a positive correlation between interns’ self-rated and observed skills in problem-solving, clinical reasoning, and crisis management. Time pressure and technical difficulties were reported during ChatGPT-4o sessions. ChatGPT-4o was judged to be an effective, safe, and accessible tool for clinical training.
Suárez-García et al. (32)/Mexico	To evaluate the effectiveness of a generative AI-driven simulation tool designed to train diagnostic communication skills in medical students.	Prospective, single-arm, pre–post interventional study.	ChatGPT 4o	Diagnostic communication skills improved markedly after GenAI-based training, with scores rising from 49.96 to 86.70 (∆ = 36.74, *p* < 0.001, Cohen’s *d* = 2.58). Inter-rater reliability was excellent (ICC ≈ 0.90), supporting the robustness of the scoring.
Luke et al. (33)/Singapore	To evaluate the performance of ChatGPT in tutorial and case-based learning questions in physiology and biochemistry for medical undergraduates, focusing mainly on the GPT-3.5 version and comparatively assessing GPT-3.5 and GPT-4 performances in a subset.	Comparative study	ChatGPT 3.5 and 4o	GPT-3.5 performed better in physiology (74.7) than in biochemistry (59.3). GPT-3.5 struggled with clinical application of physiology and produced less precise biochemistry explanations, while GPT-4 outperformed GPT-3.5 in both subjects. GenAI performance varies by discipline and question type.
Sridharan and Sequeira, (34)/Kingdom of Bahrain	To evaluate how generative AI tools can create learning outcomes, test items, and assessment standards for teaching hypertension treatment in undergraduate medical education	Observational cross-sectional study	Sage Poe, Claude-Instant, ChatGPT 3.5	GenAI tools produced specific learning outcomes correctly aligned with Bloom’s taxonomy action verbs. ChatGPT added explanatory answers that supported self-study. The Objective Structured Clinical Examination items were appropriate for the learner stage and included relevant pharmacotherapeutic considerations.
Gim et al. (35)/Australia and the United States	To evaluate the feasibility of using a large language model (LLM) to support interactive case-based learning by responding accurately to medical student questions following a patient case script	Cross-sectional pilot study	ChatGPT 4o	The LLM produced 857 question–response pairs, adhering to the script in 97.1% of cases (832/857). Beyond the script, 96% of additions (24/25) were medically appropriate. Overall accuracy was 99.9% (856/857 appropriate responses).
Lopez and Goh. (36)/Singapore and Mexico	To use ChatGPT to create culturally sensitive case-based learning scenarios for medical students, helping them address the needs of diverse patient populations and improve communication skills	Proof of concept/application research.	ChatGPT	ChatGPT can produce culturally sensitive educational cases, but the quality of output depends heavily on prompt clarity and specificity. Potential cultural biases were noted. GenAI still struggles with complex sociocultural nuance.

The articles reviewed cover different aspects of integrating GenAI into case-based learning (CBL) in medical education, organized into six specific thematic areas discussed below.

### Theme 1: AI-mediated case interaction and its association with clinical reasoning outcomes

Across the included studies, GenAI was implemented in case-based learning through several distinct integration points (22–36). In most cases, large language models (e.g., ChatGPT-4, GPT-3.5, DeepSeek, Claude) served as interactive virtual patients, real-time reasoning support tools, automated formative feedback systems, or AI-assisted grading mechanisms (25, 27, 31, 35). Learners engaged with GenAI through structured dialogue, iterative prompt–response exchanges, case-simulation scenarios, or the submission of written case analyses for automated scoring (25, 31, 32, 35). In some studies, GenAI provided stepwise scaffolding by prompting learners to justify diagnostic hypotheses, interpret investigations, or refine management plans (22, 24, 33). In others, it simulated patient responses during standardized encounters, requiring students to gather history, formulate differentials, and respond dynamically to evolving clinical information (31, 32). These varied implementation approaches provide important context for interpreting the reported reasoning outcomes.

ChatGPT-4o showed strong alignment with expert assessments of clinical reasoning, with a high correlation between AI and expert ratings in a study conducted in Australia (25). Medical students worked through five neurological cases (meningitis, radial nerve palsy, giant cell arteritis, Ménière’s disease, and myasthenia gravis) online, with each case reviewed by four student investigators and scored independently by clinical experts using predefined criteria. Artificial Intelligence scoring showed strong agreement with expert evaluations [*r* = 0.67–0.99; interclass correlation coefficient (ICC) = 0.78–0.82]. In a collaborative study between institutions in Australia and the United States (35), a large language model (LLM) served as a virtual patient responding to neurological case simulation scenarios. The models adhered to 97.2% (832/857) of the scripted cases, and 96% of their additional responses were judged medically appropriate and by expert reviewers. These findings suggest that when GenAI is used as an interactive reasoning scaffold or a virtual patient, it may support structured diagnostic thinking; however, the heterogeneity of implementation methods limits definitive conclusions about causality. As case complexity increased, the model adjusted its reasoning depth to guide the stepwise development of clinical competence.

Consistently, a mixed-methods study from Turkey used ChatGPT-4o as a virtual standardized patient to develop complex clinical encounters for sixth-year medical students (31). Through interactive dialogue, students practiced case management, diagnostic reasoning, and clinical communication. The author reported improvements in clinical reasoning, noting that GenAI interaction encouraged a more systematic review of patient information, a more precise formulation of clinical questions, and better decision-making under time pressure. In a separate experimental study from China (22), students in the GenAI-supported CBL group achieved higher scores than those taught through traditional methods (77.3 ± 4.3 vs. 66.5 ± 5.4, respectively; *p* < 0.05). This improvement was linked to AI-generated explanations, which helped students connect biochemical concepts to their clinical relevance and strengthen conceptual integration. However, students reported less interaction with instructors compared to the traditional method of instruction (mean = 7.17/10) and rated GenAI less favorably for complex reasoning (mean = 4.2/10) and innovative thinking (mean = 3.90/10).

A comparative study from Singapore (33) found that GPT-4 exhibited stronger reasoning coherence and greater contextual accuracy than GPT-3.5 in physiology and biochemistry assessments. GPT-4 provided more logically structured and contextually suitable answers, especially in biochemistry, indicating enhanced performance. However, both models still occasionally produced factual inaccuracies, highlighting the risk of plausible but incorrect statements even in advanced systems. Similarly, a quasi-experimental study from China (24) cautioned against overreliance on GenAI tools, as it revealed several hallucinations in the form of factual inaccuracies.

### Theme 2: Efficiency and scalability of case creation

Generative AI substantially reduced the time required to complete case-based learning tasks. Students in the AI-CBL group finished their assignment more quickly than those in the traditional group (2.6 h vs. 5.5 h, respectively; *p* < 0.05), suggesting that GenAI tools supported faster case analysis and problem-solving (22). In clinical biochemistry tasks, experts noted that GenAI produced comprehensive responses by integrating emerging biomedical themes, including changes in the tumor microenvironment, epigenetics, and multidisciplinary treatment strategies. Additionally, participants in the AI-CBL group reported higher examination scores than those in the traditional CBL group (77.3 ± 4.3 vs. 66.5 ± 5.4, respectively; *p* < 0.05). A proof-of-concept study from Singapore and Mexico (36) further demonstrated GenAI’s creative and cultural adaptability in medical education. Through iterative prompt refinement, researchers showed that ChatGPT could create culturally sensitive case scenarios. Initial screening revealed stereotypical elements, but adding cultural, religious, and socioeconomic details made the cases more inclusive and contextually appropriate. The final AI-generated cases covered diverse socio-cultural contexts, including language barriers, religious practices, and economic challenges. Likewise, a feasibility study from Australia (27) confirmed that LLM outputs are factually accurate and reliable for preoperative medical training. The model answered 543 out of 547 questions correctly (99.3%), with all responses validated by an intensive care trainee and a medical education consultant. The four unanswered items showed the model appropriately declined to make up information, and no hallucinations were found after manual review; occasional additions beyond the script were medically accurate. This method also accelerated case development by allowing experts to improve AI-generated drafts, and the free-text query feature supported self-directed learning outside faculty hours.

In parallel, GPT-4o provided reliable automated formative feedback and scoring, demonstrating high agreement with expert ratings and suggesting potential to streamline grading processes (25). Agreement with expert ratings was high (*r* = 0.67–0.90), and a 10-point calibration was applied to offset the model’s tendency to score 12 percentage points higher, aligning GenAI scores with expert evaluations. A single-arm interventional study from Mexico (32) demonstrated that AI can generate unscripted, on-demand patient interactions for diagnostic communication practice, resulting in significant improvements in communication scores, with mean performance score increasing by 36.7 points and proportion of high-performing students rising to 70%, highlighting the scalability, cost-effectiveness, and usefulness for repeated, individualized skill development.

### Theme 3: Learner engagement, motivation, and perceived usefulness

Students participating in AI-assisted anatomy and physiology CBL reported higher engagement, satisfaction, and self-directed learning than those in traditional CBL, as measured by structured Likert-scale survey instruments developed within each study context (23, 29). These instruments primarily assessed perceived engagement, clarity of feedback, motivation, and self-directed learning behaviors. Although the studies reported internal consistency and alignment with instructional objectives, most relied on researcher-developed survey tools rather than previously standardized psychometric instruments. Consequently, the findings reflect learner perceptions in specific educational settings rather than outcomes derived from externally validated measurement frameworks. In an observational cross-sectional study from Bahrain (34), three GenAI tools (Sage Poe, Claude-Instant, and ChatGPT-3.5) were incorporated into pharmacology and therapeutics teaching on hypertension management. Expert review confirmed that these tools produced contextually relevant materials designed to support critical thinking and conceptual understanding. They were also seen as valuable tools for educators, capable of creating high-quality test questions and engaging teaching materials, as validated by a structured content validation framework. A feasibility study from the United Arab Emirates (30) similarly found that AI-generated cases encouraged learner reflection and maintained engagement, supporting GenAI as an effective complementary CBL resource. Further evidence from a quasi-experimental study in China (23) showed improved learner motivation and intention to continue engagement when DeepSeek was integrated into problem-based learning for hematology residents. The DeepSeek-assisted group recorded higher ratings on a five point Likert scale (1 = Strongly disagree to 5 = Strongly agree) in effective course analysis (4.40 ± 0.56 vs. 3.97 ± 0.67, respectively; *p* = 0.009), intention to continue using AI (4.47 ± 0.51 vs. 3.63 ± 0.62, respectively; *p* < 0.001), trust in AI output (4.47 ± 0.51 vs. 4.03 ± 0.62, respectively; *p* = 0.004), clarity of feedback (4.53 ± 0.51 vs. 3.73 ± 0.64, respectively; *p* < 0.001), and understanding of medical concepts (4.33 ± 0.55 vs. 3.87 ± 0.63, respectively; *p* = 0.003).

Similar trends were reported in biochemistry CBL, where students, through a survey, rated GenAI-integrated sessions as more interactive, time-efficient, and participatory (22). Ease of use was rated highly (mean = 9.50 on a 1–10 scale), reflecting minimal technical barriers. Perceived usefulness of GenAI tool, measured through questionnaire on a 0–10 scale, increased from 5.5 to 7.6 (*p* < 0.05), reflecting students’ appreciation of the tool’s improved content quality and efficiency. Artificial intelligence was also rated highly for perceived knowledge acquisition (Mean = 9.18) and the provision of Supplementary materials (Mean = 6.47), positioning it as a valuable complement to textbooks and e-learning platforms. Students frequently described GenAI as a “non-judgmental learning partner” that encouraged curiosity and sustained inquiry (35). The model showed 99.9% compliance with case scripts, with deviations verified as medically appropriate by expert reviewers, highlighting its reliability in maintaining scripted case integrity while allowing medically appropriate variation. A pilot study from the United Kingdom (29) examining GenAI use within technology-enhanced CBL for second-year medical students found self-reported improvements in clinical reasoning and communication, measured using Likert-based self-report tools. Students in the GenAI-supported group reported higher engagement and greater confidence in applying clinical knowledge, with 70% of students in the GenAI-CBL group describing it as a supportive, low-pressure aid for self-directed learning.

### Theme 4: Accuracy, reliability, and ethical concerns

The integration of GenAI into case-based learning (CBL) has demonstrated strong accuracy and reliability, although several ethical issues remain. In preoperative medical education, AI-generated answers showed 99.3% factual accuracy, with no hallucinations reported after manual comparison with curated case scripts using predefined criteria (27). When uncertain, the model declined to respond to 4 out of 547 questions, reflecting a conservative response strategy rather than fabricating information; however, these omissions were still considered inappropriate because the correct details were available in the script. Accuracy varies across LLM models. A comparative analysis showed that GPT-4 produced more coherent reasoning and higher-quality answers in physiology and biochemistry assessment than GPT-3.5, as judged by expert examiners using standardized schemes; however, occasional factual errors persisted (33). GenAI tools were found capable of synthesizing recent medical evidence and addressing limitations of standard resources in specific domains. Zeng et al. (24) found that ChatGPT provided up-to-date information on retinitis pigmentosa, incorporating recent advances in gene therapy and optogenetics that went beyond traditional sources. However, its accuracy decreased in more complex areas, especially in electroretinogram interpretation, where it made errors and struggled to integrate multiple clinical concepts.

Faculty noted that AI-generated case studies often exhibited strong structural consistency, but limitations persist in areas that require novel case synthesis, advanced clinical reasoning, and contextual adaptation. These included difficulty drawing analogies across similar cases and insufficient depth when explaining complex clinical concepts (24). Beyond authenticity and coherence, these observations highlight the importance of clinical safety and error tolerance when AI-generated cases are used in medical education. For example, a medical biochemistry study employed a hepatocellular carcinoma case in which the AI tool generated multiple treatment options, including immunotherapy combinations, targeted therapies, and stem cell–based approaches (22). In such a high-risk clinical context, subtle inaccuracies in diagnostic reasoning, treatment options, or interpretation of investigations, particularly during prognosis discussions or treatment planning, may reinforce unsafe clinical assumptions. Thus, careful attention to clinical validation processes, such as systematic expert review, verification against evidence-based guidelines, and predefined acceptability thresholds, is essential to ensure educational safety. In such contexts, the role of GenAI is best confined to supporting case development and reflective reasoning, rather than functioning as an authoritative clinical source.

Bias and cultural sensitivity also emerged as additional concerns. Lopez and Goh (36) reported that, although ChatGPT expedited the development of culturally diverse scenarios, initial outputs occasionally exhibited stereotypical or biased assumptions, exemplified by depicting a “typical” 65-year-old woman as married with two children. These patterns highlight inherent biases within the training data and underscore the importance of meticulous prompt formulation, iterative refinement, and proactive educator oversight to achieve inclusive representation. Action research from the United States by Lundgren et al. (26) raised further concerns about data transparency and potential bias in AI-driven clinical-uncertainty simulations. Without clear disclosure of model training data, users cannot assess where biases originate or how they might influence diagnostic cues. Missing demographic information may reinforce stereotypes, influencing learners’ reasoning in problematic ways. The authors recommended transparent data disclosure, systematic bias audits, and formal ethical review to safeguard the integrity of AI-supported learning.

### Theme 5: Faculty adaptation and pedagogical integration

Faculty played a central role in adapting and integrating generative AI and immersive technologies for medical education. In ethics-approved pilot programs, AI platforms, VR headsets, and AI-generated case prompts were integrated into small-group CBL sessions under faculty supervision to ensure curricular alignment and address technical limitations (29). Iterative design cycles, involving repeated rounds of designing, testing, feedback, and refinement, helped keep AI-supported cases relevant and responsive to learner needs (26). In pharmacology teaching, Sridharan and Sequeira (34) found that although GenAI produced high-quality test items and case materials aligned with core learning objectives, Bloom’s taxonomy, clinical relevance, and learner level, faculty review using a structured content-validity framework was still essential to preserve conceptual accuracy. Faculty thus served as curators of AI-generated content, ensuring academic rigor. A commentary from Australia by Stretton et al. (28) similarly described ChatGPT’s value in developing CBL materials, producing assessment items, and generating gamified scenarios. ChatGPT could simulate various clinical roles and patient interactions, enabling educators to design adaptive, responsive learning environments. Such creative integration may offer additional opportunities to strengthen engagement and experiential learning.

Generative AI was also applied to grading and evaluation tasks, primarily to support grading consistency and objectivity, with potential implications for faculty workload. Li et al. (22) found no significant differences between GenAI-based and instructor-based grading of student presentations, indicating that AI-supported assessment can align closely with expert judgment. However, reduced reliance on instructors for routine evaluation was associated with fewer student–instructor interactions during assignments, underscoring the need for faculty to maintain active communication and mentorship to support learning processes. Variations in faculty confidence and AI literacy also affected the quality of integration. Hou et al. (23) found that uneven training and limited familiarity with GenAI tools hindered effective use in PBL settings, underscoring the importance of adequate preparation for both instructors and students. Faculty adaptation was particularly evident in diagnostic communication training. Suárez-García et al. (32) reported that AI-driven virtual patient systems were able to simulate clinical encounters and provide real-time feedback, reducing the need for continuous, hands-on faculty supervision during routine practice interactions. Importantly, this did not compromise learning quality; rather, it reconfigured the faculty role toward higher-level oversight, calibration, and facilitation, consistent with the need for ongoing educational and ethical supervision. The authors emphasized the need for careful configuration of AI systems, including safety settings to maintain clinical accuracy, pedagogical appropriateness, and alignment with instructional goals.

### Theme 6: Hybrid and ethical-reflective learning models

Hybrid AI–human learning environments integrating generative AI with virtual reality (VR) are believed to enhance reflective learning and promote ethical awareness in medical education, as evidenced by student reflections and feedback from their use of these technologies (29). These immersive settings were perceived as safe spaces for practicing clinical communication and history taking, enabling students to apply clinical knowledge more effectively. Limitations such as circular dialogue, in which the GenAI repeatedly returned to similar topics without advancing the clinical scenario, and socially desirable but medically implausible responses that lacked clinical accuracy prompted students to reflect on their communication style and the ethical implications of their decisions. Lundgren et al. (26) similarly found that ChatGPT and Claude.ai facilitated ethical reasoning in uncertainty-based clinical cases. Ethical reflection was evaluated through learner feedback and analysis of discussions/interactions, where AI-mediated discussions motivated learners to examine bias, responsibility, and decision-making, thereby enhancing ethical reflection in complex scenarios. GenAI was also used to generate culturally responsive case variations, incorporating language cues such as limited English proficiency, socioeconomic details such as uninsured migrant workers, and religious considerations such as fasting, which were reported to broaden ethical reflection and promote more inclusive discussions in global CBL settings (36).

In ophthalmology education, GenAI prompted students to reflect on ethical boundaries such as patient safety, risks of misinterpretation, and the need for expert validation (24). While learners valued rapid evidence synthesis, they stressed the importance of human oversight to prevent overreliance and maintain sound clinical judgment. These concerns are particularly important in high-stakes specialties such as oncology, where ethical decision-making extends beyond diagnostic accuracy to emotionally and morally complex domains. In such contexts, the use of AI-generated simulations to support conversations such as disclosing unfavorable diagnoses, estimating prognosis, or discussing eligibility for clinical trials raises distinct ethical challenges. Simulated responses that oversimplify uncertainty, inadequately convey empathy, or present prognostic estimates with unwarranted confidence may inadvertently normalize ethically inappropriate communication practices. As a result, AI-CBL scenarios require carefully defined boundaries, explicit faculty moderation, and structured debriefing to ensure that sensitive discussions remain aligned with professional standards, patient-centered values, and ethical norms. Rather than replacing clinician judgment, AI simulations in these settings are best positioned as prompts for guided reflection on communication strategies, uncertainty management, and ethical responsibility.

Structured reflection exercises were reported to enhance analytic depth. Luke et al. (33) reported that reviewing and critiquing ChatGPT-generated responses by engaging students in analyzing AI answers helped students develop evaluative skills by identifying strengths and limitations in the model’s reasoning. This process supports elements of deep learning and may enhance critical thinking skills. Although GPT-4 showed improved coherence and reasoning over GPT-3.5, flawed outputs, such as inaccurate physiological applications, inadequate biochemical explanations, or contradictory statements, highlighted the need for guided critique to promote critical evaluation and ethical reasoning. Collaborative reflection between faculty and students also enhanced CBL materials. Joint review of AI-generated test items helped ensure clinical accuracy, alignment with evidence-based guidelines, and ethical accountability through thorough faculty oversight that maintained adherence to treatment guidelines and protected fairness and integrity in the learning materials used for student assessment and instruction (34).

## Discussion

This scoping review aimed to map and synthesize current evidence on how generative artificial intelligence is being incorporated into case-based learning (CBL) in medical education. It examined the ways GenAI tools are used, their effects on learner engagement, the ethical issues they raise, and the limitations reported across studies. To our knowledge, this is one of the earliest reviews to focus comprehensively on the use of GenAI in CBL. Earlier research on AI in education has centered on analytics, adaptive learning systems, and automated tutoring, with limited attention to the emerging use of GenAI for case creation and instructional design. Current evidence suggests that GenAI is starting to influence how CBL is created and implemented in medical education. Its use extends beyond improvements in clinical reasoning and teaching efficiency to include effects on student motivation and emerging ethical considerations, driven by applications such as automated case generation, real-time feedback, and the redistribution of faculty effort toward facilitation and mentorship.

Generative AI is emerging as a cognitive scaffold that reshapes how clinical reasoning develops within case-based learning (CBL). Rather than serving solely as a source of information, it functions as an interactive environment that supports knowledge building through structured, real-time feedback. Alignment between expert evaluations and AI-generated grading suggests that GenAI can provide reliable formative assessment of clinical reasoning, rather than replace expert judgment (25). Through timely, structured feedback, students can iteratively review and refine their reasoning across repeated case attempts, which may support deeper engagement and progressive mastery. Combining this automated feedback with faculty oversight makes integration of GenAI into CBL more impactful, as it creates an ecosystem that effectively nurtures clinical reasoning development (23). High adherence to scripted clinical scenarios and medically appropriate responses across tasks such as history-taking, examination, and investigation further demonstrates the positive contribution of GenAI integration, particularly in supporting diagnostic and clinical management decisions (35). By allowing learners to examine the strength of their diagnostic decisions through its feedback process, GenAI appears to stimulate metacognitive awareness in CBL. The assessment approaches used across the included studies influence how reported learning gains should be interpreted. Knowledge-based quizzes and short-term pre–post comparisons suggest that certain improvements may reflect recall or task familiarity rather than durable understanding (29, 34). In contrast, rubric-guided evaluations and simulation-based clinical reasoning tasks indicate movement toward deeper learning, as these formats require application and contextual judgment (22, 32, 35). However, the lack of longitudinal follow-up limits confidence in whether these gains represent sustained, transferable clinical reasoning.

Öncü et al. (31) observed that during time pressure and cognitive strain, AI-mediated dialogue encouraged self-assessment, recognition of reasoning gaps, and resilience, which is central to reflective thinking. Generative AI also supports integrative cognition by linking concepts across biomedical and clinical domains, such as biochemistry, pathophysiology, and diagnosis, reducing cognitive load through structured case breakdown (22, 24). This integration allows learners to connect the underlying mechanism with the presenting symptoms, interpret findings more coherently, and generate more accurate diagnostic hypotheses. Furthermore, advanced GenAI models strengthen logical coherence and conceptual organization. Luke et al. (33) noted that GPT-4 produced deeper reasoning and more precise factual responses than GPT-3.5, supported learners in forming clearer diagnostic pathways and making more accurate clinical decisions. While this structured integration is educationally valuable, it also raises concerns about learners’ overdependence, limiting independent hypothesis generation and reducing the productive uncertainty essential for authentic clinical problem-solving (24). At the same time, GenAI-supported CBL has been described as encouraging more interactive and iterative engagement with clinical cases, aligning conceptually with constructivist models of experiential learning.

It is important to distinguish between the types of cases for which GenAI provides meaningful educational support. Knowledge-based cases, which emphasize factual recall, structured clinical reasoning, evidence synthesis, and stepwise diagnostic pathways, appear well-suited to AI-enhanced CBL. In these contexts, GenAI can serve as an effective cognitive scaffold by providing timely feedback, organizing information, and supporting iterative reasoning. In contrast, skill- and attitude-based cases, which require nuanced communication, emotional sensitivity, ethical judgment, and professional identity formation, present limitations for AI-supported learning. While GenAI may facilitate reflection through simulated dialogue or feedback prompts, these competencies continue to depend on human mentorship, contextual understanding, and authentic interpersonal interaction. Explicitly recognizing this distinction helps prevent overextension of GenAI use and supports its responsible integration within competency-based medical education.

The efficiency and scalability within case-based learning (CBL) have been transformed with the integration of generative AI, aligning it with foundational models in instruction design theory, such as ADDIE (Analysis, Design, Development, Implementation, and Evaluation) and the resource-based learning framework (37). By automating core instructional tasks such as case generation, feedback, and assessment, GenAI streamlines course preparation. For instance, the tool significantly reduces case discussion preparation time from 5.5 h to 2.6 h (22), while maintaining a comparable or even superior performance outcome (mean AI score: 82.2% vs. expert score: 70.3%) (25). Automated grading and adaptive feedback of GenAI also strengthens the development and evaluation stages of instructional design, producing assessments that align closely with expert judgment and maintain high fidelity to case scripts (27). Within a resource-based learning perspective, GenAI can broaden pedagogical access to educational materials by enabling the rapid generation of varied and customizable content, supporting alignment with curricular aims and learner readiness (36). This scalability enables rapid adaptation of CBL across specialties, languages, and cultural settings, an important advantage in resource-constrained curricula. While these efficiencies decrease faculty workload, they also raise concerns about excessive automation. Overreliance on GenAI can produce superficial content and limit educators’ active role in shaping case complexity, contextual nuance, and ethical guidance (22, 36). Therefore, ongoing faculty involvement in co-designing, reviewing, and refining AI-generated materials is crucial to maintaining academic quality and ensuring that efficiency improvements do not compromise educational standards.

Learner motivation and affective engagement also emerged as an important dimension of AI-supported CBL. Consistent with Self-Determination Theory (SDT), immediate feedback, flexible case exploration, and opportunities for self-paced reasoning were identified to strengthen students’ sense of autonomy and competence (22, 23). In GenAI-supported groups, students frequently reported greater confidence and satisfaction, attributing these gains to timely and clear feedback that reduced their dependence on instructors (23, 29). Iterative interaction with the model also allowed students to review and refine their responses, thereby improving reasoning and diagnostic accuracy (34, 35). This pattern is consistent with SDT’s internalization pathway, in which external guidance can encourage intrinsic motivation and mastery (38). Some studies observed a novelty bias, in which students’ enthusiasm was linked more to the newness of the technology than to its lasting educational benefits (23). This suggests that part of the motivational and emotional response to AI-CBL may stem from initial excitement rather than from more lasting engagement. Reduced collaborative dialogue in GenAI-supported sessions also raised concerns about relatedness, a core SDT component important for peer interaction and emotional engagement (29). This further indicates that, although GenAI-CBL enhances autonomy and competence, maintaining motivation depends on balancing algorithmic guidance with active mentorship to ensure meaningful engagement.

Ethical reasoning, cultural representation, and faculty readiness were the most important themes that shaped the educational value of GenAI integration in CBL. Close alignment between GenAI- and expert-graded assessments (22) and evidence of improved clinical reasoning (24) further point to its instructional reliability, though simplification of complex issues remains a recognized limitation (29). Despite high factual accuracy and low rates of hallucinations, some AI-generated cases include subtle cultural or demographic stereotypes, highlighting the need for culturally responsive prompt designs and routine bias mitigation review (36). Ethical development progressed as students critically examined GenAI outputs, reflected on discrepancies, and analyzed reasoning pathways, enhancing their analytical skills and precision. Studies involving uncertainty-based cases and AI-mediated patient dialogue revealed that engaging with ambiguous information increased accountability and critical appraisal, leading learners to justify their decisions and evaluate the reliability of GenAI guidance (26, 29). Faculty readiness is crucial in this process; educators need to assess AI-generated cases, recognize cultural and ethical issues, and mentor students effectively. Without proper oversight, efficiency improvements could unintentionally increase bias, skew clinical expectations, and reduce ethical reflection. These issues highlight that GenAI is not just a technical tool but also a pedagogical influence that transforms the teaching of clinical judgment, cultural sensitivity, and ethical reasoning.

**Figure 2:**
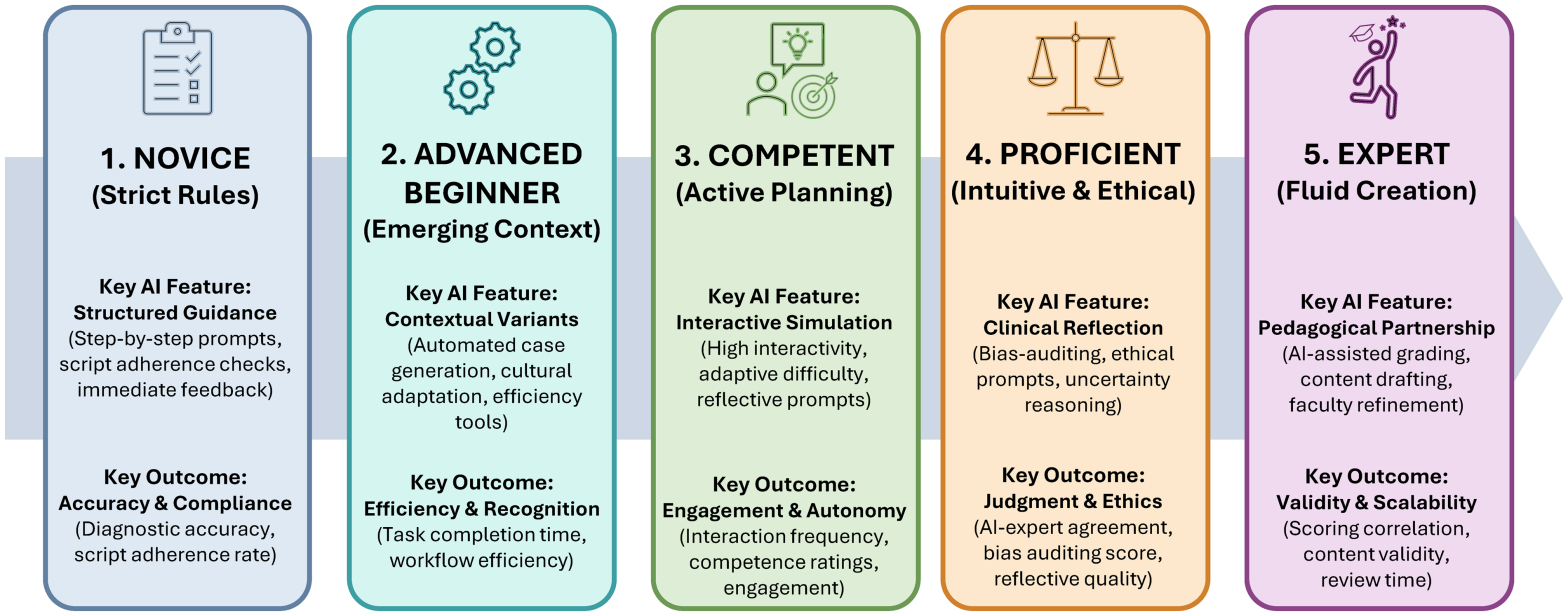
Alignment of key themes of the scoping review with the five Dreyfus stages. The figure progresses from Novice to Expert. Most of the findings align with stages 1–3 of the Dreyfus model.

The conceptual analysis of the alignment of AI-BL with the Dreyfus Model of Skill Acquisition (39) showed that, so far, GenAI has been effectively used in supporting case-based learning at early stages (*Novice* to *Competent* levels) of medical education (Figure 2; Supplementary Table 2). These include stepwise prompting, script-adherence checks, immediate feedback, automated case generation with cultural adaptation, and high levels of interactivity with adaptive difficulty. Fewer studies corresponded to the higher stages (*Proficient* and *Expert*) of the Dreyfus model, where GenAI was mostly limited to supporting functions, including assisted grading, bias auditing, and engagement with uncertainty-based reasoning prompts. This creates opportunities for future studies to apply GenAI-supported CBL optimally and/or equally across all Dreyfus stages.

### Limitations

This scoping review has some limitations that should be considered when interpreting the findings. The included studies varied widely in design, outcomes assessed, and reporting quality, but this is inherent in scoping review, and the studies were appropriate in mapping emerging evidence. Also, the Dreyfus model was used as a conceptual lens to interpret patterns across studies rather than as a detailed, stage-by-stage empirical assessment of learner progression. From an evidence-based perspective, most research has focused on early- or intermediate-level educational outcomes, leaving significant gaps in understanding how GenAI supports higher-order skill development.

## Conclusion

This scoping review analyzed current evidence on the use of generative artificial intelligence in case-based learning in medical education. The thematic analysis highlights that generative AI functions more as a learning partner than as a mere automation tool. These findings also emphasize the ongoing importance of faculty oversight and pedagogical intent in AI-supported learning. Future research should explore how GenAI can enhance advanced clinical judgment and ethical reasoning, thereby promoting sound clinical decision-making, ethical integrity, and professional thoughtfulness, while maintaining key ethical principles. Further investigation into the continued role of faculty oversight in AI-enabled educational settings is also necessary.

### Research gaps and future directions

This review highlights several key research gaps and methodological limitations that need thorough investigation. While generative AI-supported case-based learning shows promise, the current evidence base is methodologically limited and focused on early development. First, most studies are pilot, feasibility, quasi-experimental, or single-arm with small sample sizes. There is a need for randomized controlled trials, multi-center comparative studies, and longitudinal research to assess whether AI-supported case-based learning produces lasting improvements in diagnostic reasoning, knowledge retention, and clinical performance. Second, outcome measures often focus on short-term exam scores, Likert-scale perceptions, and self-reported confidence. Future studies should include validated clinical reasoning instruments, workplace assessments, OSCE performance metrics, and measures of transfer to real clinical settings. Instruments should be supported by multiple sources of validity evidence, including content, internal structure, response process, and consequences, to enhance the interpretation of results related to affective, engagement, and reasoning aspects. Third, most studies target Novice and intermediate learners. Research should explore AI-supported case-based learning at advanced levels, especially concerning uncertainty management, hypothesis generation, diagnostic calibration, and professional identity development. Fourth, ethical and epistemic issues are underexplored. Future work should investigate automation bias, cognitive offloading, overreliance on algorithms, and changes in epistemic authority in AI-supported learning. Structured bias audits and culturally sensitive evaluation methods are needed to ensure fairness and ethical standards. Finally, research on implementation is vital to the sustainable integration of competency-based medical education. This encompasses faculty training, governance, policy development, cost-effectiveness, and strong evidence of validity for AI assessment tools. Addressing these priorities is crucial to advancing AI-supported case learning from initial innovation to evidence-based, ethical educational practice.

## Data Availability

The original contributions presented in the study are included in the article/Supplementary material, further inquiries can be directed to the corresponding author.
