# Supplementary material for: AI-supported case-based learning in medical education: a comprehensive scoping review
Source: Front Med (Lausanne). 2026 Mar 25;13:1798097. doi: 10.3389/fmed.2026.1798097 (PMC13056847; doi:10.3389/fmed.2026.1798097)
Supplement: Supplementary file 2 [file Data_Sheet_2.pdf]

Supplementary Table 1: Methodological details of studies included in the Scoping review.

References/ Country	Methodology
Li et al. (18) / China	The control group used the traditional method of CBL delivery while the experimental group used an AI-assisted approach. The performance of the two groups was assessed independently by teachers and AI tools.
Hou et al. (19) / China	Control group completed the hematology courses using the traditional PBL approach, while the experimental group used DeepSeek-assisted PBL with real-time AI guidance. Both groups covered the same case content, and their performance and feedback were assessed through exams and questionnaires.
Zeng et al.(20)/ China	The experimental group completed the RP review using an AI-assisted approach, while the control group used the standard self-directed review method. Both groups received the same lecture content and followed the same review framework, with individual study time monitored and no collaboration allowed.
Qian et al. (21)/ United States	Students engaged in neurological case-based learning via an AI-assisted online platform. Their interactions were scored by an LLM and compared against expert evaluations, with calibration applied to align the scores
Lundgren et al. (22)/ United States	The study combined empirical data, generative AI case drafting, and continuous collaborative refinement with medical educators and students to create adaptive case-based learning resources that support learners in navigating clinical uncertainty.
Trewren et al. (23)/ Australia	The study used generative AI to draft five expert-refined perioperative cases, delivered via a custom GPT-4o platform for interactive patient simulations, supporting learners in clinical uncertainty.
Stretton et al. (24) / Australia	Commentary.
Hassoulas et al.(25) / United Kingdom	The study employed a technology-enhanced learning (TEL) approach, integrating immersive facilities, virtual reality (VR), and generative AI-simulated virtual patient cases to facilitate medical students' practice of clinical skills and exploration of 3D anatomy. Efficacy was assessed through quizzes and qualitative feedback.
Berbenyuk et al.(26) / United Arab Emirates	Two COVID-19 clinical cases were generated using OpenAI ChatGPT 4.0 and reviewed by experts. This was presented to a workshop where medical educators used a predefined assessment framework, rating statements on a scale of 1 to 5 to assess their quality and educational value
Oncu et al.(27) / Turkey	Medical interns interacted with ChatGPT-4o as a virtual standardized patient to assess their clinical case management skills, combining self-assessments, observer evaluations, interviews, and technical observations.
Suárez-García et al.(28) / Mexico	Medical students completed T2DM diagnostic simulations: a pre-test with ChatGPT, AI-based training with ten more ChatGPT simulations, and a post-test with human standardized patients. Blinded raters used an 8-domain rubric for assessment
Luke et al. (29)/ Singapore	GPT-3.5 generated answers for physiology and biochemistry modified essay questions (MEQs). Two subject experts independently graded each response using a standardized marking scheme. Subsequently, a subgroup of MEQs from each subject had answers generated by GPT-4, which were also graded by the same experts
Sridharan and Sequeira, (30) / Kingdom of Bahrain	The AI tools generated specific learning outcomes and various test items focused on systemic hypertension pharmacology and therapeutics, designed for both pre-clerkship and graduating medical students. The authors independently evaluated the AI-generated outputs for accuracy and relevance.

Gim et al. (31)/Australia and the United States	Large Language Model (LLM) was utilized to present patient scenarios in a question-and-answer format for case-based learning (CBL). Its performance was assessed by evaluating its responses to medical student questions with the aim of deriving a diagnosis and formulating a plan.
Lopez and Goh.(32) /Singapore and Mexico	Iterative prompts were used to create culturally sensitive educational cases for medical students. Outputs were evaluated for diversity, learning objectives, and bias.

Supplementary Table 2: Mapping of AI-supported CBL themes to Dreyfus stages of skills acquisition.

The table displays Dreyfus stages, typical learner behaviors at each stage, alignment of AI-CBL themes with each stage, corresponding AI features, suggested metrics for measuring outcomes, and implementation recommendations.

**Dreyfus Stage**	**Typical Learner Behaviors**	**AI-supported CBL Themes**	**Concrete AI feature**	**Suggested Outcome Metrics**	**Notes/ Implementation ideas**
**Novice**	Follows explicit rules, limited pattern recognition.	1, 2, 3	Structured prompts, script adherence, and virtual patient simulation; rapid case generation, and corrective feedback; short interactive dialogues for clarification.	Percentage diagnostic accuracy, completeness of reasoning steps, time to reach diagnostic hypothesis, cognitive load rating, and script adherence rate.	Use tightly structured single-diagnosis cases, offer an immediate retry option after errors, and employ AI-generated checklists to make each reasoning step explicit and transparent to learners.
**Advanced Beginner**	Recognizes recurring aspects; begins applying context.	1, 2, 3	Pattern recognition, contextual and cultural customization, automated case variants, iterative feedback on common errors, and adaptive difficulty.	Case preparation and completion time, ratings of contextual/cultural appropriateness, change in recurring-error frequency, and perceived workflow efficiency.	Compare multiple AI-generated variants of the “same” case and identify the changes; iteratively refine cases with added contextual details; automate low-level tasks to allow learners to focus on recognizing cues and context.

**Competent**	Plans actions, manages multiple aspects, and prioritizes.	1, 2, 3, 4	Complex reasoning and prompting alternate diagnosis; Adaptive difficulty; Iterative feedback and self-paced exploration	Interaction frequency, task completion rates with increased difficulty, competence ratings (e.g., Likert-based rating), perceived clarity and usefulness of feedback, and pre/post reasoning-quality scores	Use AI-driven feedback for targeted revisions, schedule self-paced AI-supported cases followed by faculty debriefs, design revision cycles where learners revisit prior AI cases at increased difficulty while comparing their reasoning traces longitudinally, and include basic AI limitation feedback.
**Proficient**	Intuitive grasp of situations; recognizes patterns.	4,6	Reflective reasoning, bias- and hallucination-detection; ethical reasoning,	Percentage of accurate AI-responses/ Hallucination rate, AI–expert agreement for complex cases; bias-audit scores, performance on structured ethical-reasoning tasks	Introduce subtle AI errors or biased suggestions for critique; require brief ethical reflection summaries after complex cases; conduct cultural-bias audits on AI outputs; explore decision thresholds in ambiguous cases.
**Expert**	Fluid, tacit knowledge; rapid pattern matching.	4,5,6	AI-assisted drafting of complex case series, and grading rubrics; bias and uncertainty detection.	Expert-AI scoring correlation, faculty time saved, content validity rating, and documentation of ethical/professional guidance given around AI use.	Generative AI drafts initial cases and assessments for expert refinement; pair AI-assisted grading with experts' calibration; use analytics to spot cohort reasoning gaps and redesign teaching

Themes: (1) Clinical reasoning support; (2) efficiency and scalability of cases; (3) learner engagement, motivation, and perceived usefulness; (4) accuracy, reliability, and ethical concerns; (5) faculty adaptation and pedagogical integration; and (6) hybrid and ethical-reflective learning models.
